# Platelets and platelet-derived factors in curcumin treatment: a narrative review of mechanisms and translational potential

**DOI:** 10.1515/med-2026-1430

**Published:** 2026-04-27

**Authors:** Dandan Li, Yuxin Huo, Mutalifu Xiaheidan, Xiaotong Liu, Difeng Wu, Zihua Liu

**Affiliations:** The Second Hospital of Lanzhou University, Lanzhou, China; School of Basic Medical Sciences, Central South University, Changsha, China; The Second Clinical College of Lanzhou University, Lanzhou, China; School of Veterinary Medicine and Biosafety, Lanzhou University, Lanzhou, China; The First Clinical Medical College of Lanzhou University, Lanzhou, China; Department of Blood Transfusion, the Second Hospital of Lanzhou University, Lanzhou, China

**Keywords:** curcumin, platelets, platelet-derived factor, action pathway

## Abstract

Based on the characteristics of curcumin, such as low bioavailability, low toxicity, wide availability, and multiple biological activities, and in conjunction with its established pharmacological effects on anti-platelet activation, aggregation inhibition, and platelet count enhancement, the following hypothesis is proposed: Curcumin may exert therapeutic effects in multiple systems, including cardiovascular, neurological, digestive, musculoskeletal, and genitourinary systems, by modulating platelet function and its derived factors, thereby interfering with various disease-related pathways. In light of this, the present study aims to address the following questions: Through which specific molecular pathways and mechanisms does curcumin regulate platelets and their derived factors, thereby exerting therapeutic effects on diseases in different systems? Additionally, this research seeks to provide a theoretical foundation for further exploration of novel therapeutic directions for curcumin in disease treatment.

## Introduction to curcumin and platelets

Curcumin is a polyphenolic compound with low bioavailability and rapid metabolism, which requires combination with other compounds to enhance its bioavailability and clinical application value [1], 2]. In this study, curcumin was commonly combined with other compounds for platelet-targeted delivery to treat various diseases. Curcumin is widely used as a traditional medicine in countries such as India and China, and it is effective in the prevention and treatment of various diseases [2]. Current research has confirmed that curcumin has low toxicity, broad availability, and low cost, and possesses multiple pharmacological properties including disinfection, analgesia, anti-inflammatory, antioxidant, anti-fibrotic, anticancer [5], antiplatelet activity, anti-angiogenesis, promotion of cell apoptosis, and wound healing [1]. It is recommended for the treatment of skin diseases such as eye infections, burns, bites, and acne, as well as digestive system disorders like diarrhea, indigestion, excessive stomach acid, bloating, and ulcers, and has even been proposed as an antidepressant [2]. Since the continuous exploration of curcumin’s medicinal value, its physiological and pharmacological effects have become increasingly well understood, but many aspects remain unexplored and require ongoing research [1].

Platelets are anucleate cell fragments derived from megakaryocytes. They have a lifespan of 5–7 days and play a central role in hemostasis, coagulation, thrombosis, and tissue repair. Additionally, they exhibit immunomodulatory and tumor metastatic effects [6]. Research demonstrates that vascular injury triggers platelet activation through the interaction of surface receptors (e.g., GPIb-IX-V complex) with vascular wall adhesion proteins and soluble agonists. This process releases bioactive molecules including ADP and thromboxane A_2_ (TXA_2_), initiating a cascade reaction [6], 7]. The activated platelets then form temporary hemostatic thrombi via adhesion, activation, and aggregation, while secreting multiple bioactive factors such as PDGF, TGF-β, VEGF, EGF, IGF, PF4/CXCL4, PDEGF, CTGF, etc. These factors collectively promote coagulation, tissue repair, and inflammatory regulation [7], 8]. Research reveals that platelet activation, aggregation, and factor release involve multiple signaling pathways, including GPIb-IX-V adhesion receptor signaling, ITAM signaling, pattern recognition receptor signaling, soluble platelet agonist-induced G protein-coupled receptor signaling, integrin inside-out/in signaling, arachidonic acid pathway, platelet cGMP signaling, and PI3K-Akt signaling. These pathways interact through coordinated actions of ADP, TXA_2_, thrombin, phospholipase C (PLC), P-selectin, and Ca^2+^, forming an intricate network that drives robust platelet responses [9]. The relationship between inflammation and platelet function is complex. In inflammatory diseases, abnormal platelet activation and functional changes exacerbate inflammatory responses. Studies demonstrate that platelets, through interactions mediated by selectins and their ligands, β2-integrins, and ICAM-1, collaborate with neutrophils in inflammatory processes, particularly in vasculitis [10].

Platelets are closely associated with inflammatory factors and immune responses. Abnormal platelet function can trigger various diseases, not limited to thrombotic disorders [80]. Firstly, it is well-established that endothelial injury can induce platelet activation and coagulation system activation. Overly activated platelets contribute to the pathological formation of occlusive arterial or venous thrombi [81], which may lead to conditions such as myocardial infarction (MI), acute ischemic stroke, or venous thromboembolism (VTE) [82]. Additionally, thrombosis is observed with numerous clinical conditions, including cancer, systemic inflammation (e.g., sepsis), antiphospholipid syndrome (APS), immune thrombocytopenia (ITP), trauma, stent implantation, blood transfusion, liver disease, thrombotic microangiopathy (TTP, HUS, HELLP), heparin-induced thrombocytopenia (HIT), malaria, COVID-19-associated infections [83], and many other diseases [84]. Secondly, the interaction between platelets and inflammatory factors is prominently observed in inflammatory bowel disease (IBD). Patients with IBD, such as Crohn’s disease and ulcerative colitis, exhibit enhanced platelet reactivity, persistent coagulation activation, and impaired fibrinolysis. The hypercoagulable state in these patients increases the stability of thromboembolic events and reduces thrombus degradation. Endothelial dysfunction and immune-mediated disturbances in the anticoagulation pathway further exacerbate coagulation abnormalities. Furthermore, IBD during active phases often presents with thrombocytosis, likely driven by inflammatory cytokines such as IL-6 and thrombopoietin, leading to a hypercoagulable state. The binding of CD40L expressed on activated platelets to CD40 on endothelial cells promotes the upregulation of VCAM-1, ICAM-1, and IL-8. Platelet-leukocyte interactions form platelet-leukocyte aggregates, both of which contribute to persistent inflammation and endothelial dysfunction in IBD, thereby impairing mucosal healing and exacerbating gastrointestinal ischemic injury [85]. Furthermore, platelet-related, coagulation, and inflammatory reactions can lead to neuroinflammation, neurovascular dysfunction, and hypercoagulability, often contributing to common neurodegenerative diseases such as Alzheimer’s disease and Parkinson’s disease [86]. Sun S et al. summarized that various circulating substances, including autoantibodies, immune complexes, VWF, DAMPs, complement factors, and extracellular vesicles, can trigger sustained platelet activation. Activated platelets may also modulate innate and adaptive immune system functions by releasing multiple pro-inflammatory and immune mediators or through direct interactions with immune cells (e.g., B cells and T cells). These mechanisms play roles in various diseases, including idiopathic thrombocytopenic purpura (ITP), systemic lupus erythematosus (SLE), antiphospholipid syndrome (APS), drug-induced thrombocytopenia (DITP), heparin-induced thrombocytopenia (HIT), COVID-19 vaccine-induced thrombocytopenia (VITT), thrombotic thrombocytopenic purpura (TTP), and hemolysis, i.e., heparin-induced liver injury and thrombocytopenia syndrome (HELLP) [87]. The potent physiological and pathological effects of platelets are evident, and platelet abnormalities can trigger cascade reactions at multiple levels of the body, leading to various diseases. Abnormal platelet parameters also serve as concrete manifestations of numerous disorders. Given the positive effects of curcumin on platelets, utilizing platelets as a therapeutic target for related diseases has become a major focus in clinical research.

Previous studies have demonstrated that inflammatory mediators and cellular effector factors of chronic inflammatory responses are critical factors in promoting cancer initiation and progression, particularly within the tumor microenvironment (TME), which includes cancer cells, immune cells, and other components [76]. Additionally, Kuan Wang, Kejin Li, and their respective teams have proposed in colorectal cancer research that the platelet-to-lymphocyte ratio (PLR), platelet-to-albumin ratio (PAR), and blood inflammatory markers are significantly associated with patient prognosis [76], 78]. Furthermore, in lung cancer studies, it has been suggested that patients benefit from targeted therapy, although emerging challenges such as tumor gene reprogramming and immune resistance remain major obstacles [78]. Another study by Boxiang Zhang found that cancer immunotherapy often induces immune-related acute kidney injury (IRAI) [79]. Concurrently, chemotherapy-induced peripheral neuropathy (CIPN) and chemotherapy-related cognitive impairment (CRCI) have garnered increasing attention [77]. Exploring alternative therapeutic approaches to break through cancer treatment remains a current research focus and challenge. Studies have conclusively demonstrated that tumor cell-platelet interactions are essential for successful hematogenous metastasis. Tumor cells express adenosine diphosphate (ADP), thromboxane A_2_ (TXA_2_), high-mobility group box 1 (HMGB1), adhesion G protein-coupled receptor CD97, and tissue factor (TF), which interact with toll-like receptor 4 (TLR4) or activate the coagulation cascade. This process stimulates local platelet activation, enabling platelets to form thrombi that encapsulate circulating tumor cells (CTCs), thereby evading NK cell attack and clearance [11]. Meanwhile, activated platelets secrete abundant growth factors and chemokines (e.g., VEGF, PDGF, or TGF-β), which suppress NKG2D-induced NK cell dysfunction. By activating endothelial P_2_Y_2_ receptors, these platelets facilitate the extravasation of tumor cells into distant organs through subendothelial stroma, while stimulating tumor proliferation and angiogenesis, thereby promoting tumor dungrowth and metastasis [11]. Leveraging platelets’ physiological properties and their unique interactions with tumor cells, platelet-based targeted drug delivery systems have emerged as a prominent research focus, exemplified by curcumin applications [12].

## Effect of curcumin on platelet and its derived factors

Curcumin demonstrates beneficial pharmacological effects and biological activity on platelets. Its antiplatelet effects target three key mechanisms: platelet adhesion, activation, and aggregation. With demonstrated potential to inhibit thromboembolism, atherosclerosis, and leukemia treatment, it also exhibits anti-inflammatory, antioxidant, anti-fibrotic, anti-infective, and anticancer properties. Acting across multiple physiological systems, curcumin exerts therapeutic effects on various diseases [1], [12], [13], [14]. Extensive research has yielded significant findings regarding its mechanisms of inhibiting platelet activation, aggregation, and apoptosis.

**Figure 1:**
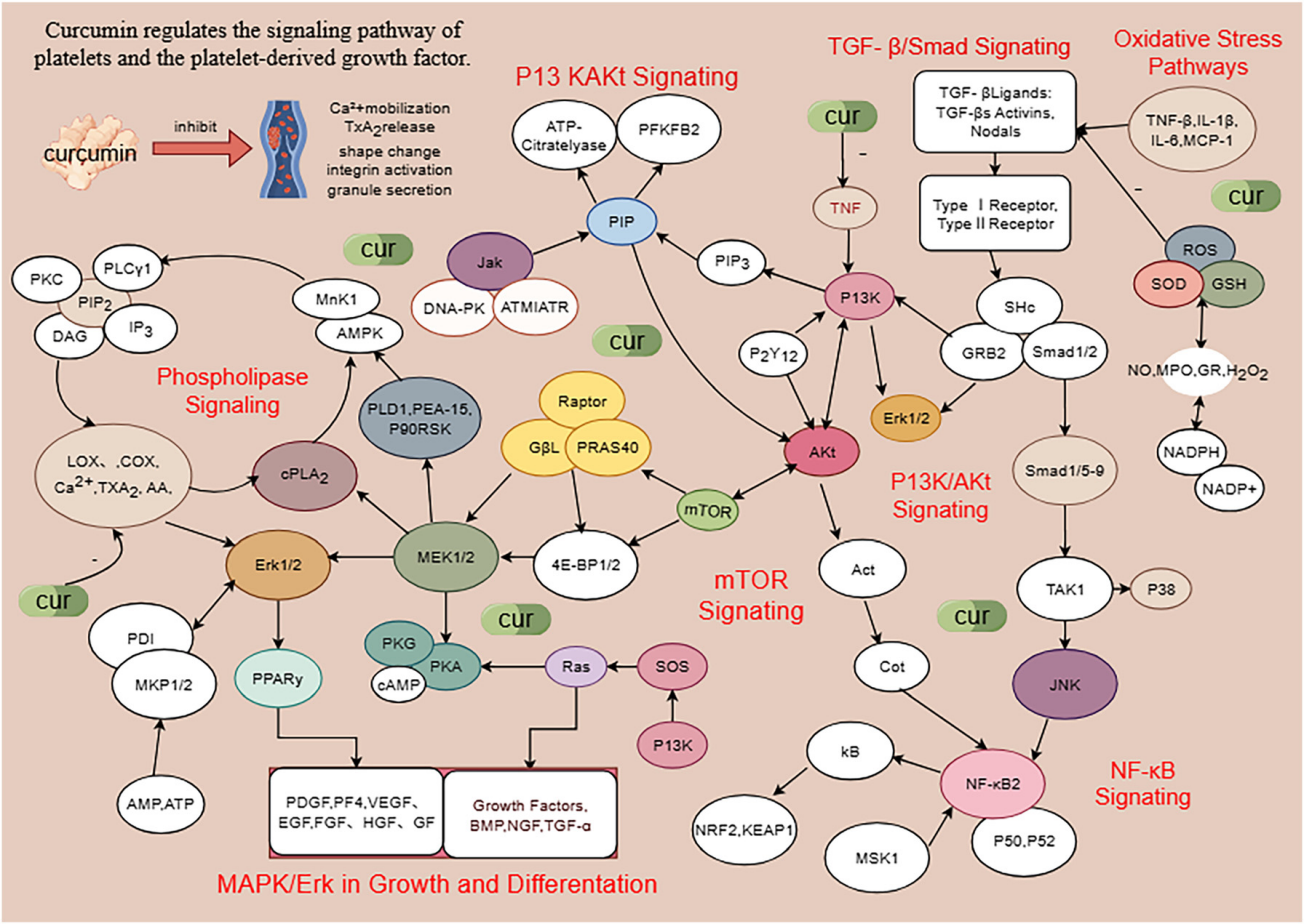
General overview of the signaling pathways by which curcumin regulates platelet activation and platelet inhibition [1], [13], [14], [15], [16], [17]. curcumin can regulate platelet activation, aggregation and apoptosis by regulating Ca^2+^ mobilization, TXA_2_ release, granule secretion, integrin and enzyme activation. Cur, curcumin; PDGF, platelet-derived growth factor; VEGF, vascular endothelial growth factor; PPAR-γ, peroxisome proliferator-activated receptor; ErK1/2, extracellular signal-regulating kinase; JNK1/2, c-Jun N-terminal kinase; COX, cyclooxyenase; GSK, glycogen synthase kinase; JAK, janus kinase; JNK, c-Jun N-terminal kinase; MAPK, mitogen activated protein kinase; mTOR, mechanistic target of rapamycin; NF-κB,nuclear factor kappa-enhancer of activated B cells; PAR, protease-activated receptor; PDI, protein disulfide isomerase; PGI_2_, prostaglandinI_2_; PI3k, phosphoinositide 3-kinase; PKA, cAMP-dependent protein kinase; Akt, protein kinase B; PKC, protein kinase C; PKG, cGMP-dependent protein kinase; PLC, phospholipase C; ROS, reactive oxygen species; SOD, superoxide dismutase; GSH-Px, glutathione peroxidase.

Curcumin exerts extensive effects on platelets within the body, including coagulation and angiogenesis, platelet activation and aggregation, autophagy, oxidative state of platelets, as well as impacts on platelet-derived growth factors and related diseases [1], 14]. The complex interactions between curcumin and platelets involve multiple signaling pathways and regulatory factors. Key pathways include: MAPK/ErK signaling [14], 15], mTOR signaling, lipoxygenase/epoxygenase/calcium ion pathways, protein kinase C (PKC) pathway, NF-κB signaling [16], antioxidant mechanisms [15], intracellular biochemical processes, adhesion molecules and leukocytes, coagulation and fibrinolysis [13], 17]. Curcumin may also inhibit platelet function through mitochondrial membrane potential modulation [1]. Its multifaceted effects on platelets involve various factors such as: cyclooxygenase (COX), lipoxygenase, arachidonic acid (AA), thromboxane A_2_ (TXA_2)_, fibrinogen, 12-hydroperoxypentaenoic acid (12-HPETE), purinase, platelet-derived growth factor (PDGF), vascular endothelial growth factor (VEGF), peroxisome proliferator-activated receptor (PPAR-γ), extracellular signal-regulated kinase (ErK1/2), c-Jun N-terminal kinase (JNK1/2), as well as bioactive substances like P-selectin, TNF-α, and interleukins [1], 13], 14]. Studies have demonstrated that curcumin inhibits platelet apoptosis through oxidative stress and inflammatory responses [14], 17], 18]. Recent research by Kurnegala Manikanta suggests purified curcumin may serve as a potential therapeutic bioactive molecule for treating oxidative stress-induced platelet activation, apoptosis, and related complications [18], 19]. The main interaction pathways between curcumin and platelets in the body are summarized in Figure 1. In addition, *in vitro* studies showed that curcumin could inhibit platelet activation and aggregation, inhibit platelet apoptosis, and increase platelet count, which could be used as a platelet protector *in vivo* and *in vitro* [1], 14], 17].

**Figure 2:**
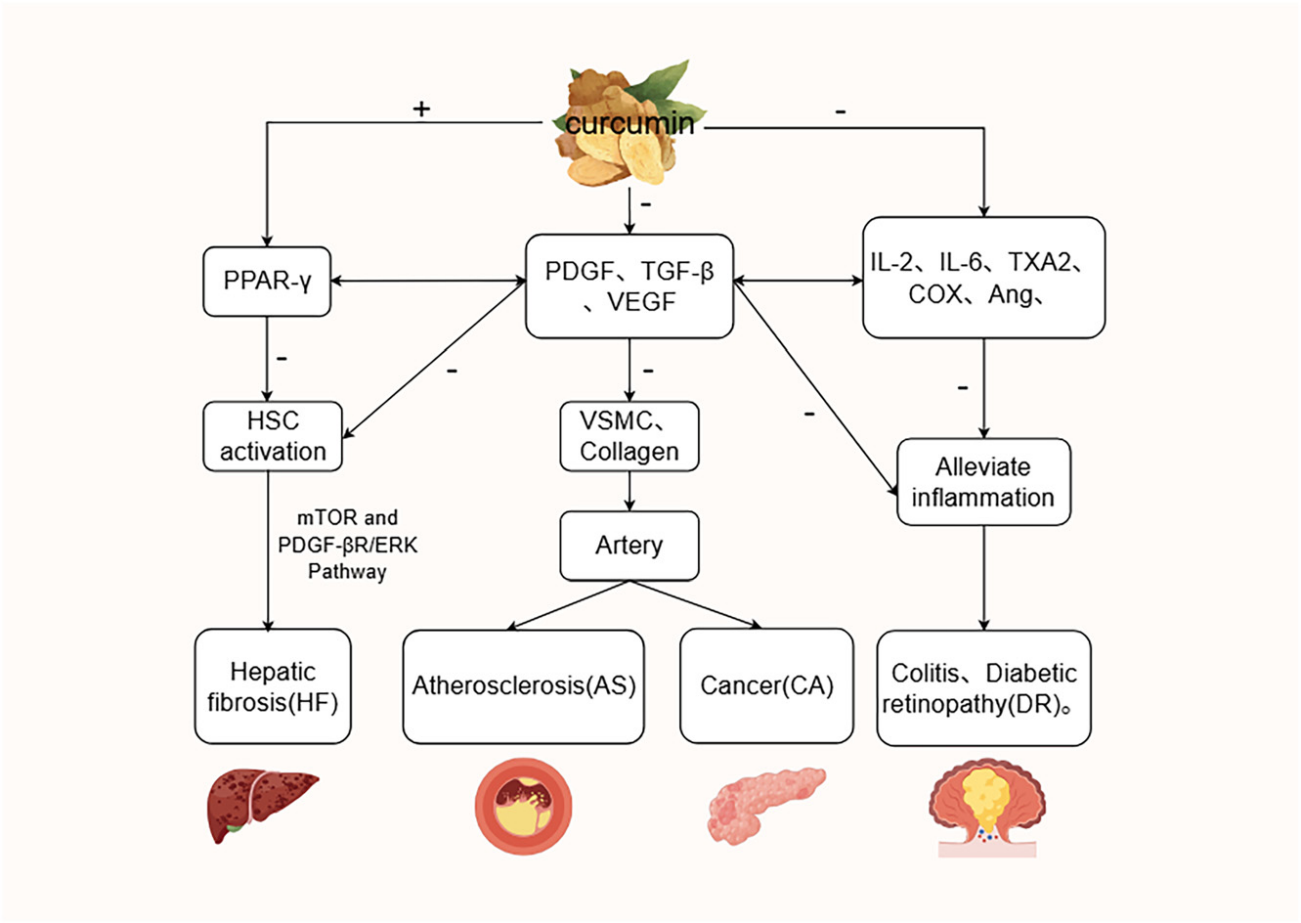
The role of curcumin in the PDGF pathway [14], 16], 20], 21].

Platelet-derived growth factor (PDGF) plays a crucial role in human physiology by regulating platelet aggregation through vascular feedback mechanisms [1]. Studies have demonstrated that PDGF overexpression is observed in liver cirrhosis and pulmonary fibrosis. Curcumin, a therapeutic anti-fibrotic agent, inhibits hematopoietic stem cell (HSC) activation-induced damage and fibrosis by increasing PPAR-γ levels and reducing the production of TGF-β, PDGF, its receptors, and type I collagen [14]. Research has confirmed that curcumin selectively disrupts the mTOR and PDGF-βR/ErK signaling pathways, effectively suppressing PDGF overexpression to influence pathological vascular formation and improve conditions such as atherosclerosis and tissue fibrosis [1], 14], 16], 20]. Additionally, Jamshid Tabeshpour’s study indicates that curcumin inhibits PDGF-induced proliferation, migration, and collagen synthesis in vascular smooth muscle cells (VSMCs), while also suppressing PDGF-mediated VSMC actin-cytoskeletal reorganization, reducing PDGF signal transduction, and blocking PDGF-receptor binding. These mechanisms play a pivotal role in regulating vascular injury responses, providing further evidence for the therapeutic potential of curcumin [14]. Subsequent studies have demonstrated that curcumin can inhibit the transcription of heat shock protein 90 (HSP90), nuclear factor κB, and hypoxia-inducible factor-1α in pancreatic cancer cells, as well as the secretion of vascular endothelial growth factor (VEGF), angiogenin 1, angiogenin 2, platelet-derived growth factor (PDGF), cyclooxygenase-2, and transforming growth factor-β (TGF-β). In xenograft tumor models, curcumin downregulates VEGF, HSP90, hypoxia-inducible factor-1α, and cyclooxygenase-2, highlighting its potent anti-angiogenic effects [14]. Curcumin exerts protective activity against early diabetic complications through the VEGF/platelet/endothelial cell adhesion molecule signaling pathway. Recent research by Mariaelena Filippelli et al. reveals that curcumin modulates pro-inflammatory factors such as IL-6, TNF-α, and IL-2, as well as angiogenic factors like PDGF-AB, thereby influencing diabetic retinopathy [21]. Additionally, studies have shown curcumin’s therapeutic effects on colitis through PDGF modulation [16]. In summary, research on curcumin-PDGF interactions has yielded significant findings, as detailed in Figure 2.

## Effect of curcumin on platelet and platelet-derived factors in the regulation of disease

In recent decades, the pivotal role of platelets in inflammation, oxidative stress, atherosclerosis, and thrombotic disorders has been established, demonstrating their indispensable function in disease pathogenesis, progression, and outcomes – particularly in cardiovascular conditions. The physiological properties of curcumin suggest its potential dual action in inflammatory disorders involving platelet dysfunction, where it may both modulate platelet function and mitigate inflammatory responses. This provides a novel therapeutic approach for rheumatoid arthritis, inflammatory bowel disease, and related conditions [1]. Based on current evidence, this review synthesizes curcumin’s anti-inflammatory and anti-platelet activation effects, while summarizing its regulation of platelets and platelet-derived factors in disease mechanisms. Specific findings are summarized in Table 1.

**Table 1:** Summary of curcumin action on disease-related pathways in various systems.

S.no.	System	Principal disease	Mechanism of action	Cite
1	Cardiovascular system	Vasculitis, atherosclerosis, hyperlipidemia, thrombosis and embolism	TXA2, CX3CR1, PDGF, cholesterol homeostasis, expression of TLR4 receptor; activation of GP IIb/IIIa receptor, etc.; signaling pathways such as TGF-β1/Smad3, JAK2/STAT3, P38-MAPK, JNK, NF-κB; levels of triglycerides and ADA expression;	[1], 3], 13], 14], [27], [28], [29, 31], 35]
2	–	Diabetic eye disease, wound healing in diabetic patients	Platelet/endothelial cell adhesion molecule signaling pathway; related to the expression of TGF-β, PDGF, VEGF, FGF2, etc.; immune system, etc.;	[37], 38], 41], 42]
3	Nervous system	AD, Parkinson’s disease, stroke, TBI, anxiety, depression, stroke, etc.	Expression of PTAFR; autophagy and apoptosis, IL10-STAT3 signaling pathway; Ras/ERK, PKC, PI3K and MAPK intracellular signaling pathways;NRF2/HO-1 pathway;	[1], 3], 30], [46], [47], [48]
4	Alimentary system	Ulcerative colitis, liver fibrosis, pancreatic cancer, liver cancer, etc.	PPAR-γ, NF-κB, p38MAPK, PI3K/AKT/mTOR, PDGF-βR/ERK, JNK, Notch signaling pathways; PDGF and CTGF/LRP1 pathways; glutathione metabolism and arachidonic acid metabolism pathways; regulating the expression levels of ALT, AST, ALP, TBIL, BAX proteins;	[14], 16], 20], 50], 51], 55], 68]
5	Kinetic system	Rheumatoid arthritis, osteoarthritis, etc.	Akt/MTOR, TLR-4/NF-κB signaling pathway; purine metabolism enzymes related in neutrophils, lymphocytes and platelets;	[56], [57], [58], [59], [60]
6	Urinary system	Diabetic nephropathy, renal fibrosis, kidney injury caused by lead poisoning, prostate cancer, etc.	TGF-β pathway; oxidative stress; PDGF related;	[52], 61]
7	Reproductive system	Menopause, dysmenorrhea, premenstrual syndrome, ovarian cancer, etc.	JAK/STAT signaling pathway; regulation of hsCRP, THBS5, etc.;	[56], 62]

### Cardiovascular diseases

Cardiovascular diseases (CVD) stand as one of the foremost health threats in contemporary society, with thrombosis serving as a critical pathological foundation for numerous cardiovascular disorders. Platelets play a pivotal role in this process [6], [7], [8]. Current clinical research has conclusively demonstrated that curcumin exhibits remarkable protective effects on cardiovascular health. This bioactive compound demonstrates therapeutic potential in multiple conditions including heart failure, myocardial infarction, coronary atherosclerosis, stent restenosis, hypertension, hyperlipidemia, diabetic cardiomyopathy, and hypertrophic cardiomyopathy [22], [23], [24]. First, curcumin can significantly reduce oxidative stress in vascular endothelial cells (VECs) by regulating multiple signaling pathways, while also modulating lipid metabolism to inhibit plaque formation. Additionally, it suppresses vascular smooth muscle cell (VSMC) proliferation, promotes angiogenesis, and exerts anti-atherosclerotic effects. Second, curcumin protects myocardial cells from ischemia-hypoxia-induced damage, inhibits apoptosis, hypertrophy, and fibrosis in cardiomyocytes, improves ventricular remodeling, and reduces drug-induced myocardial injury, thereby alleviating diabetic cardiomyopathy (DCM). Third, this study provides a detailed exposition of curcumin’s mechanisms of action, pharmacokinetics, and toxicology [22]. In summary, curcumin holds significant clinical value in the prevention and treatment of cardiovascular diseases [1], 13], 14], 22].

#### Improvement of endothelial function, atherosclerosis

Current research demonstrates that atherosclerosis pathogenesis is associated with endothelial dysfunction [25], with disease progression primarily linked to platelet aggregation and reduced vascular endothelial blood flow. Platelet activation pathways involved include the arachidonic acid pathway, adenosine diphosphate pathway, serotonin pathway, nitric oxide pathway, and free radicals [26]. Additionally, curcumin can improve endothelial dysfunction by modulating platelet function, playing a pivotal role in the development of arterial hypertension, atherosclerosis, heart failure, ischemia-reperfusion injury, Alzheimer’s disease, and other conditions [27]. The interaction between platelets and inflammation/immune responses has been explicitly demonstrated in previous studies. Zhou L et al. proposed that curcumin potently inhibits vascular inflammation by suppressing key pathways such as nuclear factor κB (NF-κB) and mitogen-activated protein kinase (MAPK), while alleviating oxidative stress through activation of the Nrf2/HO-1 axis. Curcumin also improves lipid metabolism by reducing cholesterol synthesis and promoting cholesterol reversal. Furthermore, it inhibits the proliferation and migration of vascular smooth muscle cells (VSMCs), enhances endothelial function, and stabilizes plaques [88]. Yang C et al. indicated that curcumin downregulates the expression of TLR2 and TLR4 on human endothelial cells (HECs), thereby suppressing lipopolysaccharide (LPS)-mediated HMGB1 release. By inhibiting the HMGB1/TLR/NF-κB signaling pathway, curcumin reduces the release of inflammatory factors induced by human cytomegalovirus (HCMV) infection in human umbilical vein endothelial cells (HUVECs), thereby ameliorating the progression of atherosclerosis (AS) [22]. Karimian MS et al. summarized that the anti-inflammatory effects of curcumin are attributed to reduced mRNA and protein expression of intercellular adhesion molecule 1 (ICAM-1), vascular cell adhesion molecule 1 (VCAM-1), and P-selectin, as well as modulation of NFκB, JNK, p38, and STAT-3 in endothelial cells, thereby decreasing transendothelial monocyte migration [27]. Studies have shown that curcumin decreases reactive oxygen species (ROS) production, monocyte adhesion, c-Jun N-terminal kinase (JNK) phosphorylation, p38 activation, and STAT-3 expression in human umbilical vein endothelial cells (HUVEC) stimulated by tumor necrosis factor-α (TNF-α). It also reduces mRNA and protein levels of intracellular adhesion molecule-1 (ICAM-1), monocyte chemotactic protein-1 (MCP-1), and interleukin-8 (IL-8), helping prevent adverse vascular effects of pro-inflammatory responses [27]. Research by Manzoni AG et al. demonstrated that rutin and curcumin, when used alone or in combination, can lower serum and immune cell inflammation, triglyceride levels, and atherogenic activity in hyperlipidemic rat models, demonstrating potential for atherosclerosis prevention and treatment [24], 28]. Recent studies by Flavia Fontana et al. demonstrate that curcumin exerts its effects on atherosclerosis through modulating cholesterol homeostasis, TLR4 receptor expression, and signaling pathways including P38-MAPK, JNK, and NF-κB. Multidisciplinary research has confirmed that bionic platelet envelope nanoparticles delivering anti-inflammatory curcumin exhibit promising therapeutic potential for atherosclerosis [29]. Weiqi Li et al. further propose that platelet granules play a pivotal role in atherosclerosis, with tetrahydrocurcumin potentially serving as an effective cardioprotective agent [3]. Huang HC’s experimental findings reveal that curcumin inhibits vascular smooth muscle proliferation via platelet-derived growth factor, providing additional evidence for its potential use in preventing atherosclerosis and restenosis [30]. From this perspective, curcumin exerts its anti-atherosclerotic effects through a polypharmacological mechanism, which involves influencing endothelial cells by affecting platelets, various inflammatory factors, or other substances, thereby achieving therapeutic goals.

#### Thrombosis and embolism

Platelet activation and aggregation serve as critical foundations for thrombus formation and subsequent embolization [1]. Reactive oxygen species (ROS) contribute to endothelial dysfunction, platelet activation, and coagulation abnormalities, thereby promoting thrombosis [18]. Funda Tamer, Elvira Giurranna, and colleagues demonstrated that curcumin modulates platelet signaling and reactivity, exerting effects on both thrombosis and hemostasis [4], 15]. *In vitro* studies by Weiqi Li et al. revealed that curcumin’s primary bioactive metabolite, tetrahydrocurcumin (THC), inhibits platelet aggregation by reducing cytoplasmic phospholipase A_2_ (cPLA_2_) phosphorylation, which decreases thromboxane A_2_ (TXA_2_) production. This significantly suppresses agonist-induced granule secretion in human gel filtration plates, including CD62P/CD63 expression and the release of platelet factor 4, CCL5, and adenosine triphosphate. Additionally, THC treatment downregulates the MAPK (Erk1/2, JNK1/2, and p38 MAPK) signaling pathway, further reducing cPLA_2_ activation, TXA_2_ generation, and granule secretion [3]. Recent research by Lin Xu et al. reported that P-Cur-PFP@PC, synthesized from platelet membrane-encapsulated curcumin nanoparticles (Cur-PFP@PC), enables ultrasound-guided low-intensity focused ultrasound (LIFU) targeting for therapeutic intervention in pregnancy-related venous thrombosis [31]. Furthermore, Jiang Ling et al. demonstrated that curcumin alleviates myocardial inflammation, apoptosis, and oxidative stress induced by acute pulmonary embolism (APE) through modulation of the microRNA-145-5p/IRS1 axis [32]. Meanwhile, based on platelet-based inflammatory homing and cell membrane stealth nanotechnology, Jin H designed PM@Cur-RV nanoparticles by combining coated polylactic-co-glycolic acid (PLGA) nanoparticles with platelet membrane vesicles (PMV). These nanoparticles effectively reduce pulmonary vascular permeability and lower pro-inflammatory cytokine loads, thereby inhibiting pulmonary vascular damage [33]. Additionally, Bai S et al. demonstrated that decellularized heterologous heart valves (DHV) hybridized with curcumin and other substances to engineer tissue-engineered heart valves (TEHV). This approach not only effectively suppresses the adsorption of plasma proteins, blood cells, platelets, and thrombi but also promotes endothelialization of TEHV while enabling responsive curcumin release to regulate immune responses, showing promising application potential [34]. Furthermore, Tabeshpour J et al. summarized that curcumin inhibits platelet-adhesion to cerebral microvascular endothelial cells, an effect associated with reduced expression levels of P-selectin, E-selectin, and GPIIb/GPIIIa on both platelets and endothelial cells [14]. Xu Ximing et al. reported that platelet membrane-coated curcumin-PLGA nanoparticles can promote astrocyte-neuron transdifferentiation for treating cerebral hemorrhage [35]. These studies provide substantial evidence for curcumin’s therapeutic effects in thrombotic diseases. On the basis of these studies, it is undeniable that curcumin exerts effects on pulmonary embolism, cerebral hemorrhage, cerebral infarction and other thrombotic diseases through different mechanisms of action, but it also fully reflects the characteristics of low bioavailability of curcumin, so the research in this aspect needs to be further deepened.

#### Wound healing in diabetic patients

Platelets play a crucial role in wound healing. Recent studies on curcumin’s wound-healing effects have primarily focused on oxidative stress and inflammatory responses. Reactive oxygen species (ROS), which promote platelet activation and angiogenesis, are closely linked to these processes [18], 22], 36]. Li X et al. designed a novel dual-drug-loaded *in situ* gel-forming nanoparticle/hydrogel system (EGF-Cur-NP/H) based on curcumin’s anti-inflammatory and antioxidant properties and the physiological characteristics of epidermal growth factor (EGF), significantly enhancing granulation tissue formation, collagen deposition, and angiogenesis [37]. Iqubal MK et al. discovered that natural drug delivery systems using curcumin and other natural compounds, targeting wound healing paradigms, involve platelet-derived growth factor (PDGF) and related factors [38]. Xiang-Yi Zhao et al. demonstrated that the curcumin-loaded platelet and hydroxypropyl chitosan hydrogel composite system can achieve effective hemostasis management and wound healing through photodynamic activation [39].

Given the chronic nature of diabetic wounds, recent research on curcumin’s wound-healing effects has predominantly focused on diabetes [22], 40]. Gufran H et al. demonstrated that pre-treating human adipose-derived stem cells (hASCs) with curcumin before co-transplantation with platelet-rich plasma (PRP) significantly improved wound healing in diabetic rats [41]. Ren S et al. developed a synergistic platform (CurCDs@iPRF-MA) using autologous platelet concentrate (APC) for treating diabetic chronic wounds. Notably, CurCDs@iPRF-MA regulates mitochondrial homeostasis under inflammatory conditions, activates oxidative phosphorylation (OXPHOS) pathways, and modifies the diabetic microenvironment through metabolic reprogramming. This mechanism enables macrophage phenotype modulation, ROS elimination, and angiogenesis via autologous growth factor release, thereby enhancing wound healing efficacy [42]. Curcumin’s ability to promote spinal cord injury in high-glucose models further supports its potential in wound healing, though its exact mechanisms require further investigation [43]. This demonstrates that curcumin exhibits certain positive effects in promoting wound healing, and the synergistic delivery of platelets combined with curcumin can significantly enhance therapeutic efficacy, indicating substantial exploratory potential.

### Neuronal repair and Alzheimer’s disease (AD)

As previously mentioned, common neurodegenerative diseases such as Alzheimer’s disease and Parkinson’s disease are closely associated with abnormal platelet activation and endothelial cell dysfunction [1], 22], 27], 86]. In their research, Bavarsad et al. proposed that curcumin can exert effects on diseases such as anxiety, depression [1], neuronal damage, Parkinson’s disease, multiple sclerosis, Huntington’s disease, and stroke [44]. Additionally, Rustichelli S mentioned in his study that Alzheimer’s disease (AD) is associated with the abnormal accumulation of heterogeneous Aβ peptides in brain parenchyma as senile plaques. The circulation and accumulation of Aβ peptides in cerebral blood vessels can also lead to cerebral amyloid angiopathy. Aβ is synthesized and released in platelets through the amyloid protein production pathway, which promotes platelet activation, enhances fibrin clot stability, facilitates reactive oxygen species (ROS) formation, and causes thrombosis [45]. Studies have shown that curcumin can effectively inhibit platelet aggregation induced by fibrillar amyloid peptides, an effect related to reduced intracellular signaling pathways involving PKC, PI3K, and MAPK. In contrast, curcumin shows limited inhibitory effects on thrombin and vulxin-induced platelet aggregation/activation, as well as on platelet inhibition stimulated by hemostatic agonists alone. Therefore, curcumin demonstrates selective and effective inhibitory activity against pathological stimuli (such as fibrillar amyloid peptides) in platelets [45]. Additionally, curcumin significantly increases the expression of brain-derived neurotrophic factor and cell viability while reducing apoptosis [1]. The combined use of curcumin and platelet-rich plasma (PRP) can enhance axonal regeneration in acute nerve injuries [46]. Meanwhile, Tang Chunming’s research demonstrates that a bionic anti-neuroinflammatory nanoplatform (DHCNPs) – featuring anti-inflammatory curcumin-loaded liposomes as the core and a biomimetic hybrid membrane formed by fusing platelet and neutrophil membranes – can hijack neutrophils to neutralize elevated pro-inflammatory cytokines. Through DNAse I modification, this platform degrades NETs, repairs the blood-spinal cord barrier, and promotes neuronal regeneration, demonstrating potential for targeting active neutrophil extracellular traps (NETs) and treating traumatic spinal cord injuries (SCI) [47]. Yaozhi He’s study reveals that curcumin-loaded milk-derived small extracellular vesicles (sEVs) fused with platelet membranes can reduce aging in human umbilical vein endothelial cells (HUVECs) induced by hyperglycemia/IL-1β (HG/IL-1β) through the NRF2/HO-1 pathway, thereby accelerating spinal cord injury recovery [43]. Liu Junxiu et al. identified platelet-activating factor receptor (PTAFR) as a potential biomarker for early Alzheimer’s disease (AD) diagnosis and treatment, which enhances microenvironmental conditions mediated by microglia via IL10-STAT3 signaling. Additionally, PTAFR serves as a presumed target for anti-AD compounds like curcumin, highlighting its therapeutic significance [48]. However, current research on curcumin’s platelet-related effects on the nervous system remains incomplete, and various experimental hypotheses require further validation.

### Fibrotic liver and kidney

Research demonstrates that curcumin regulates the mTOR and PDGF-βR/ERK signaling pathways, thereby modulating the levels of PDGF, PDGFRB, PPAR-γ, TGF-β, and TNF-α. This mechanism suppresses VEGF expression and activation in hepatic stem cells (HSCs), reduces sinusoidal angiogenesis during liver fibrosis, and effectively improves fibrotic outcomes [1], 14], 20], 49]. Additionally, Elzoheiry A et al. reported that curcumin/chitosan-coated green silver nanoparticles inhibit fibrosis-mediated proteins to exert anti-fibrotic effects [49]. Yunhang Zhu concluded that curcumin lowers levels of ALT, AST, ALP, TBIL, BAX protein, and liver indices in fibrosis models. It maintains hepatocyte membrane stability to reduce apoptosis, inhibits hepatic stellate cell activation and proliferation by suppressing inflammatory responses, and alleviates tissue peroxidative damage through oxygen radical scavenging, collectively exerting therapeutic effects on liver fibrosis [50]. Concurrently, Xiao Shuang et al. revealed that the curcumin derivative Curc-mPEG454 regulates PDGF-B/PDGFR-a, IL-34/CSF1R, PDGF-D/PDGFR-β, and CTGF/LRP1 pathways. This compound inhibits macrophage and hepatic stellate cell activation to reshape fibrotic microenvironments, while differentially expressed genes (DEGs) are predominantly enriched in retinol metabolism, glutathione metabolism, and arachidonic acid metabolism pathways [51]. These findings collectively indicate that curcumin exerts significant therapeutic effects on liver fibrosis, with close associations to platelet-derived growth factors.

Furthermore, prior studies have demonstrated that curcumin exhibits renal protective effects, which are closely associated with platelet activity, oxidative stress, and the TGF-β signaling pathway [49]. Additionally, research by Chadant Noonin et al. confirmed that curcumin prevents hyperglycemia-induced stimulation of renal cell secretory groups in fibroblast activation by reducing intracellular reactive oxygen species (ROS) and TGF-β secretion, thereby exerting therapeutic effects on diabetic nephropathy and renal fibrosis [52]. Lau WL’s experiment showed that dietary tetrahydrocurcumin improved renal fibrosis, proteinuria, and hypertension in chronic kidney disease (CKD) rats [53]. Earlier research by Abdel-Moneim AM et al. suggested that curcumin may alleviate blood biochemical changes and renal oxidative damage caused by Pb^2+^ poisoning [54]. However, research on curcumin’s regulation of platelets in the treatment of urinary system diseases is relatively limited compared to other systems, and further in-depth exploration is still required.

### Inflammatory diseases (colitis, arthritis)

Research has confirmed the mutual influence and interaction between platelets and inflammatory factors [10], with curcumin demonstrating significant effects on both. Studies show that curcumin and its combined formulations exhibit promising therapeutic effects on certain digestive system disorders, including oral mucosal fibrosis, pancreatic cancer, and ulcerative colitis (UC). Xu S et al. investigated how curcumin inhibits the activation of the PI3K/AKT/mTOR pathway, thereby suppressing platelet-induced intestinal microvascular endothelial cell invasion and angiogenesis [55]. Altinel Y et al. further demonstrated that curcumin regulates colitis induced by 2,4,6-trinitrobenzene sulfonic acid (TNBS) by modulating PDGF expression and the NF-κB signaling pathway [16]. These findings not only validate the feasibility of studying colitis through platelet-mediated mechanisms but also reinforce the inseparable relationship between curcumin, platelets, and inflammatory factors.

Studies have demonstrated that curcumin exhibits significant anti-inflammatory and analgesic effects on musculoskeletal disorders, particularly in patients with rheumatoid arthritis, knee osteoarthritis (OA), and synovitis [56], 57]. Research indicates that curcumin treatment markedly reduces platelet-associated inflammatory factors in rheumatoid arthritis patients. Xu Ximing’s study revealed that curcumin-PLGA (polylactic acid-hydroxyacetic acid copolymer) nanoparticles coated with platelet membranes can suppress inflammation and improve motor function [35]. Meanwhile, da Silva et al. demonstrated that curcumin-named vitamin D3 nanocapsules regulate purine metabolism enzymes in neutrophils, lymphocytes, and platelets, which may influence arthritis development [58]. Multiple meta-analyses have summarized that intra-articular injections of platelet-rich plasma (PRP) and hyaluronic acid (HA) can alleviate joint pain and enhance mobility [59], 60]. However, Shtroblia V’s study revealed that PRP shows no benefit in reducing pain or structural changes in OA patients, challenging the widely accepted short-and medium-term analgesic effects of PRP on knee osteoarthritis. This discrepancy may stem from variations in PRP preparation and administration methods, raising questions about the clinical efficacy of PRP [57]. In conclusion, curcumin may exert therapeutic effects on musculoskeletal disorders by modulating platelets and platelet-related factors. However, existing studies on PRP’s efficacy in joint pain relief remain controversial. Future research should conduct rigorous validation of its safety and effectiveness, while investigating whether curcumin can enhance PRP therapy through platelet function modulation.

### Cancer

As previously mentioned, inflammatory mediators, platelets, and cancer cells are closely associated [76]. Multiple studies have demonstrated that curcumin can induce apoptosis and suppress proliferation in cancer cells while inhibiting various cellular signaling pathways, showing anti-cancer effects against liver cancer, colon cancer, cervical cancer, pancreatic cancer, breast cancer, lung cancer, head and neck squamous cell carcinoma, prostate cancer, and brain tumors [5], 14], [61], [62], [63]. Chandra Manivannan A et al. demonstrated that CLEC-2, secreted by activated platelets, is expressed on specific tumor cell types and participates in tumor cell-induced platelet aggregation and metastasis. Studies have shown that it inhibits platelet aggregation and tumor metastasis in colon cancer by binding to tumor cell surfaces [61]. Vicente IST et al. revealed through canine experiments that curcumin exerts anti-tumor effects on splenic hemangioma by regulating the expression of vascular endothelial growth factor receptor-2 (VEGFR-2) and platelet-derived growth factor receptor-β (PDGFR-β), further supporting curcumin’s anticancer properties [64]. Additionally, Shengli Wan et al. developed a curcumin-encapsulated platelet membrane (PM)-camouflaged nanoparticle by leveraging the biomolecular interaction between P-selectin on platelet membranes (PM) and tumor CD44 receptors, offering a promising solution for cancer-targeted therapy [65]. Subsequent studies revealed that a novel curcumin-loaded platelet membrane bioinspired chitosan-modified liposome (PCLP-CUR) could enhance curcumin release, improve pharmacokinetic characteristics, enhance tumor targeting, and demonstrate anti-cancer effects, showing significant efficacy against liver cancer [66], 67]. Furthermore, Mozzi et al. demonstrated that curcumin binds to PDGFRα. Inactivation of PDGFRα (overexpression and/or abnormal activation) can inhibit multiple cancers, including liver, pancreatic, prostate, ovarian, and breast cancers, by blocking downstream signaling pathways that regulate cell proliferation, migration, and angiogenesis [68], 69].

Research by Yoysungnoen B et al. demonstrated that tetrahydrocurcumin can modulate the expression of hypoxia-inducible factor-1α and vascular endothelial growth factor (VEGF) in cervical cancer cell lines of nude mice, which are key to angiogenesis induction [70]. Concurrently, He GF et al. established that curcumin-DPT effectively inhibits proliferation of the cervical cancer cell line Me180 by suppressing protein expression of Notch1, NF-κB, and VEGF, thereby blocking the Notch signaling pathway [62]. Additionally, studies indicate that curcumin reduces the levels of oncogenic kinases PAK1 and PAK4, thereby decreasing melanin production to achieve whitening effects or exerting therapeutic effects on melanoma [71]. Kim MW et al. synthesized curcumin-containing red blood cells (RBCs) and platelet membranes coated with gold nanostars (R/P-cGNS), which were evaluated for their ability to release under near-infrared irradiation. These R/P-cGNS nanoparticles were shown to target melanoma cells and exert immunomodulatory effects on macrophages [72]. Furthermore, nano-curcumin, as a novel megakaryocyte stimulant, may improve chemotherapy-induced thrombocytopenia in mice through ERK1/2 and JNK signaling pathways [73]. It is also noteworthy that studies such as Hu R have summarized the extensive use of thrombopoietin (TPO-RA) in the treatment of thrombocytopenia, while its application in immune thrombocytopenic purpura (ITP) has become a major research hotspot and trend [75]. However, current studies have not yet clarified the differences in mechanisms of action, therapeutic areas, and research trends between curcumin and TPO-RA. Additionally, no definitive comparative analysis has been conducted on the similarities and differences between the two in the treatment of ITP. Further research could explore this issue in depth. In summary, leveraging curcumin’s pharmacological properties, the physiological characteristics of platelets, and their interactions with tumor cells offers potential for targeted cancer therapy. This approach also highlights curcumin’s low bioavailability as a therapeutic consideration [12]. At the same time, many unknown questions require further investigation.

### Other diseases

Curcumin demonstrates broad therapeutic effects through complex and interconnected pathways that influence multiple human physiological systems. Research by Qin Yuwen et al. reveals its potential to improve blood rheology, inhibit platelet aggregation, prevent thrombosis, and exhibit anti-inflammatory, antitumor, and anti-fibrotic properties, thereby promoting blood circulation and alleviating amenorrhea and dysmenorrhea [56]. Studies have also demonstrated significant correlations between serum hsCRP, WBC, MPV, and NLR levels with premenstrual syndrome (PMS) and menstrual pain. Notably, Talebpour A et al. confirmed curcumin’s ability to significantly reduce hsCRP in young women, potentially improving PMS and dysmenorrhea. However, the limited efficacy in clinical trials may be attributed to short follow-up periods and single-dose administration [74]. Additionally, curcumin-DPT has been shown to effectively block the Notch signaling pathway and inhibit proliferation in cervical cancer cell line Me180 [62]. Current research indicates curcumin’s therapeutic potential in reproductive system disorders, though further investigation is required to clarify its platelet-mediated mechanisms and the precise relationship between its reproductive system effects and platelet function.

## Conclusion and prospect

This study found that curcumin, as an endogenous and exogenous platelet protector, can inhibit platelet activation and aggregation, regulate platelet release responses, and increase platelet count through multiple mechanisms. Combined with its anti-inflammatory and antioxidant pharmacological effects, it can modulate various bioactive factors, effectively preventing and treating diseases related to platelets and platelet-derived factors in multiple systems, including cardiovascular, neurological, digestive, urinary, musculoskeletal, and reproductive systems, thereby reducing disease mortality. [1], 9]. The aforementioned studies also demonstrate the feasibility of curcumin-regulated platelet therapy in disease treatment. Given curcumin’s low bioavailability and unique pharmacological properties, recent research has predominantly focused on curcumin combined with platelet-targeted delivery, which holds significant future research value [1], 7]. However, it should be noted that this study specifically emphasizes the integration of curcumin with platelets as a therapeutic bridge. Current research has validated the feasibility of this approach, while studies on the PDFG pathway have been more comprehensive and conclusive. Nevertheless, research on mechanisms and outcomes of other pathways remains incomplete, indicating substantial unexplored areas for further investigation. Additionally, the findings on curcumin and platelets are fragmented and require further integration. More clinical trials are needed to investigate its pharmacokinetics and pharmacodynamics *in vivo*, as well as to validate its safety and effectiveness in treating platelet-related diseases. With ongoing research advancements, curcumin is poised to become a novel drug or adjunct therapy in the treatment of platelet-related disorders.
